# A New Property of Bromide of Potassium

**Published:** 1868-03

**Authors:** 


### A New Property of Bromide of Potassium.

Dr. Alexander J. Stone of Boston, announces a new property in Bromide of Potassium—"the power of checking the Reflex Nausea induced by the administration of Anæsthetics." He claims that nausea is the almost universal effects of ether, and then relates thirty cases where it was either prevented or relieved by the administration of thirty or forty grains of this drug.

His cases *appear* to sustain his conclusions, and still it remains for more extensive trial to prove them correct. His observations were made by the advice and partially under the direction of Prof. H. R. Storer, and his cases are well related.

Bromide of potassium is having its "benefit," and must yet suffer the reputation of doing a great many very remarkable things, and after having attained a desirable reputation, possibly, will sink into almost complete forgetfulness, something like its countless predecessors.

It is not well to be too skeptical in the effects of remedies, neither is it wise to accept as wholly true, what may appear true of their medicinal properties. There is hardly a single drug in the whole *materia medica* really having the properties they are represented to possess, and this fact should place us on guard in all our experiments to determine the effects of medicines. Ether does not in our experience "invariably" produce nausea and vomiting. If given, as in Doctor Stone's cases, after fasting, it more rarely produces this effect, and if patients vomit and thus empty the stomach, nausea and vomiting often cease from that time without the bromide of potassium. We most heartily thank Dr. Stone for the suggestion, and accept it, subject however to future trial. Before writing down as one of the reliable effects of this remedy, "a power to check reflex nausea after anæsthesia," indeed before positively saying that it possesses many of the properties now attributed to it, we would respectfully suggest that its effects be carefully noted in greater number of cases. It is said to have worked wonderful cures on all the diseases of the nervous system, both functional and organic. It is prescribed with as little discrimination as any drug was ever given, and properties attributed to it as varying and unreal as were ever attributed to any medicine. We believe it has some valuable remedial properties, but we do not believe that it has half as many as some suppose.

